# Egg-Phosphatidylcholine Attenuates T-Cell Dysfunction in High-Fat Diet Fed Male Wistar Rats

**DOI:** 10.3389/fnut.2022.811469

**Published:** 2022-02-02

**Authors:** Jessy Azarcoya-Barrera, Bethany Wollin, Hellen Veida-Silva, Alexander Makarowski, Susan Goruk, Catherine J. Field, René L. Jacobs, Caroline Richard

**Affiliations:** Department of Agricultural, Food and Nutritional Science, University of Alberta, Edmonton, AB, Canada

**Keywords:** high-fat diet, phosphatidylcholine, immunology, obesity, egg

## Abstract

Obesity is associated with immune dysfunction including an impaired T-cell function characterized by a lower IL-2 (proliferation marker) production after stimulation. Phosphatidylcholine (PC), a form of choline mostly found in eggs, has been shown to beneficially modulate T-cell responses during the lactation period by increasing the production of IL-2. To determine the impact of egg-PC as part of a high-fat diet on immune function we randomly fed male Wistar rats one of three diets containing the same amount of total choline but differing in the form of choline: 1—Control low fat [CLF, 10% wt/wt fat, 100% free choline (FC)]; 2— Control high-fat (CHF, 25% wt/wt fat, 100% FC); 3— PC high-fat (PCHF, 25% wt/wt, 100% PC). After 9 weeks of feeding, rats were euthanized. Cell phenotypes and *ex vivo* cytokine production by splenocytes stimulated with phorbol 12-myristate 13-acetate plus ionomycin (PMA+I), lipopolysaccharide (LPS) and pokeweed (PWM) were measured by flow cytometry and ELISA, respectively. Rats fed the PCHF diet had a lower proportion of CD3+ cells when compared to both the CLF and the CHF. Following PMA+I stimulation, splenocytes from the CHF group produced less IL-2 and TNF-α compared to CLF and PCHF groups. No significant differences in cytokine production were found among groups after LPS and PWM stimulation. Our results show that feeding a high-fat diet impairs T-cell responses, as measured by *ex vivo* cytokine production, which can be attenuated by providing egg-PC.

## Introduction

Obesity is defined as an excessive accumulation of body fat that increases the risk of developing other chronic diseases (1). Adipocyte hypertrophy is common in obesity and leads to hypoxia and cell death both contributing to an increase recruitment of immune cells within the adipose tissue (2). This is associated with a shift from M2-like macrophages exerting anti-inflammatory properties to M1-like macrophages secreting pro-inflammatory cytokine (i.e., TNF-α and IL-6) that contribute to a state of chronic low-grade systemic inflammation commonly documented in obesity. Many studies have shown that, both in humans and obese rodent models, obesity impairs immune cell function and is associated with poor response to vaccination (3–9).

Our group has previously reported that individuals with obesity and type 2 diabetes (T2D) have additional immune dysfunction when compared to normoglycemic BMI-matched individuals. Indeed, individuals with obesity and T2D had a lower production of IL-2 (proliferation marker), IL-6 and TNF-α by peripheral blood mononuclear cells (PBMCs) stimulated with phytohaemagglutinin, a T-cell mitogen. Similarly, in Wistar rats fed a cafeteria diet, Lamas et al. showed a lower IL-2 production by splenocytes stimulated with Concanavalin A (ConA, another T-cell mitogen) along with a reduction in the number of T helper cells (10). Therefore, Wistar rats fed a diet-induced obesity appear to be a relevant model to study the obesity-related immune dysfunction in humans.

Choline is an essential nutrient needed for a wide variety of processes in the body (11–13). Choline can be found in the diet as water-soluble forms [free choline (FC), glycerophosphocholine (GPC) and phosphocholine] and lipid-soluble forms [phosphatidylcholine (PC), sphingomyelin (SM)] (14). The two forms of choline mostly consumed in North America are PC and FC (15). Although PC can be found in a variety of foods (soy, dairy, nuts, etc.), meat and eggs are the major sources of PC in the diet (15, 16). PC plays an important role in cell membrane integrity, intestinal barrier function (i.e., mucus) and assembly and secretion of lipoproteins (17).

Studies from our group have consistently reported that feeding diets containing a higher proportion of PC (relative to FC, the standard form of choline in rodent diet) beneficially modulates immune function during both the lactation period in dams and suckling and weaning periods in offspring (18–20). Specifically, feeding a maternal diet containing 100% egg-PC led to a higher IL-2 production after T-cell stimulation (ConA) compared to a diet containing 100% FC in splenocytes of suckled pups. We further demonstrated *in vitro* that incubating splenocytes with lyso-PC, the form of PC readily available to cell, was associated with an increase proliferation rate and IL-2 secretion (18). This suggests that PC can positively modulate T-cell function and could potentially counteract some of the obesity-related immune dysfunctions.

Therefore, the main objective of this study was to determine the impact of feeding egg-PC on immune function in the context of a high-fat diet (HFD) when compared to a low-fat and a high-fat control diets containing only FC. We hypothesized that feeding egg-PC will attenuate obesity-related immune dysfunction by increasing IL-2 production after T-cell stimulation.

## Materials and Methods

### Animals and Diets

All animal care and experimental protocols were conducted in accordance with the Canadian Council on Animal Care guidelines and approved by the University of Alberta Animal Ethics Committee. Three-week-old male (*n* = 6) Wistar rats were obtained from Charles River Laboratories (Montreal, Quebec, Canada) and were housed 2 rats per cage in a temperature and humidity-controlled environment with a 12/12-h reversed light cycle. During the first week acclimatization period, rats were fed standard rat chow (Lab diet 5001; PMI Nutrition International, Brentwood, MO, USA). At 4 weeks of age, rats were randomized to one of three experimental diets all containing 1.5 g of total choline/kg of diet: Control-low fat (CLF, 100% FC), Control-high fat (CHF, 100% FC), PC-high fat (PCHF, 100% PC). Diets were fed *ad libitum* for the duration of the study (9 weeks). Before the end of the study, one rat (on the PCHF diet) died from complications unrelated to the study protocol.

The three experimental diets all contained the same amount of total choline differing primarily in the form of choline provided. The CHF and the PCHF diets were matched for the macronutrient content (50% fat, 23% carbohydrates, and 27% protein) whereas the CLF diet was composed of 20% fat, 53% carbohydrates and 27% protein. The nutrient composition of the experimental diets is presented in Table 1. Even though the total content of fat was different between the low-fat and high-fat diets, the fatty acids composition of the three experimental diets were matched closely using a mixture of oils (Table 2). The fat mixture added to the rodent diet was composed of flax oil, lard, corn oil and PC from egg (for PCHF only; Lipoid E 80). Lipoid E 80 is a phospholipid extract from egg containing about 85% PC+LysoPC, 9.5% phosphadityl-ethanolamine and 3% sphingomyelin (Lipoid GmbH, Frigenstr. 4 D-67065 Ludwigshafen, Germany). All diets met the essential fatty acid requirements for rodent and had similar polyunsaturated fatty acid (PUFA)/saturated fatty acid (SFA) and n-6/n-3 PUFA ratios. Diets were prepared weekly and stored at 4°C until fed. Feed cups were weighed and replaced every 2–3 days to prevent oxidation. Dietary intake and body weights were monitored regularly throughout the intervention.

**Table 1:** Composition of experimental diets.

**Ingredient (g/Kg diet)^a^**	**CLF diet**	**CHF diet**	**PCHF diet**
Casein	261.9	261.9	261.9
Corn starch	273.9	123.9	125.6
Sucrose	200	200	200
Vitamin mix (AIN-93-Vx)^b^	19	19	19
Mineral mix^c^	50	50	50
Calcium phosphate dibasic	3.4	3.4	3.4
Inositol	6.3	6.3	6.3
Cellulose	80	80	80
L-cystine	1.8	1.8	1.8
Choline bitartrate	3.7	3.7	0
**Fat mixture**			
Total fat mix	100	250	250
Flax oil	10	25	25
Corn oil	40	100	99
Lard	50	125	112
Egg-PC^d^	0	0	14

a *All ingredients were purchased from Harlan Teklad (Indianapolis, IN, USA), with the exception of the dietary oils that were all purchased from Safeway (Edmonton, AB, Canada)*.

b *AIN-93-VX Vitamin mix (21)*.

c *Bernhart–Tomarelli salt mixture (22)*.

d *Egg-PC was purchased from Lipoid GmbH. CLF, control low-fat; CHF, control high-fat; PCHF, PC high-fat; PC, phosphatidylcholine*.

**Table 2:** Fatty acid composition of experimental diets^a^.

**Fatty acid**	**CLF diet**	**CHF diet**	**PCHF diet**
	**(% of total fatty acids)**		
14:0	0.72 ± 0.0	0.76 ± 0.0	0.69 ± 0.04
16:0	20.3 ± 0.68	19.4 ± 0.83	20.6 ±1.34
16:1	1.14 ± 0.12	1.24 ± 0.16	1.17 ± 0.16
C18:0	10.5 ± 1.47	10.8 ± 1.54	11.5 ± 0.02
C18:1	33.3 ± 0.63	33.9 ± 0.41	33.6 ± 0.09
C18:2n6	28.3 ± 1.90	28.4 ± 0.78	27.8 ± 1.14
C18:3n3 (ALA)	5.64 ± 0.57	5.47 ± 0.65	4.79 ± 0.34
Total SFA	31.6 ± 2.15	30.9 ± 0.71	32.7 ± 1.40
Total MUFA	34.4 ± 0.74	35.2 ± 0.57	34.8 ± 0.07
Total PUFA	33.9 ± 1.32	33.9 ± 0.13	32.5 ± 1.48
Ratio n-6/n-3	5.06 ± 0.85	5.24 ± 0.76	5.80 ± 0.17
Ratio PUFA/SFA	1.08 ± 0.12	1.09 ± 0.03	1.00 ± 0.09

a *Analysis of the fatty acid composition of the three experimental diets collected weekly (n = 2 batches); ALA, α-linolenic acid; MUFA, monounsaturated fatty acids; n, omega; PUFA, polyunsaturated fatty acids; SFA, saturated fatty acids; CLF, control low-fat; CHF, control high-fat; PCHF, PC high-fat; PC, phosphatidylcholine*.

### Lipid Analysis

The fatty acid composition of the three experimental diets was measured by gas chromatography (GC). Briefly, total lipids from diets were extracted using the Folch method (23), saponified, and then methylated with hexane and BF3 (boron trifluoride). For the liver, a modified Folch method was used to extract total lipids and phospholipids as previously described (24, 25). Fatty acid methyl esters were separated and identified by automated GLC (GLC7890A; Agilent Technologies) on a 100-m CPSIL 88 fused capillary column (100 m × 0.25 mm; Agilent) as described previously (26).

### Tissue Collection and Immune Cell Isolation

At 13 weeks, male rats were weighed and euthanized by CO_2_ asphyxiation in the morning hours. Spleens were collected aseptically, weighed, and immune cells were isolated for further processing. Isolation of immune cells from the spleen have been previously described (27). Briefly, single cell suspensions were obtained by disrupting tissue through a nylon mesh screen in sterile Krebs-Ringer HEPES buffer with bovine serum albumin (5 g/l; Sigma-Aldrich Canada Ltd., Oakville, ON, Canada). Ammonium chloride lysis buffer (155 mM NH4Cl, 0.1 mM EDTA, 10 mM KHCO3; Fisher Scientific, Edmonton, AB, Canada) was used to lyse erythrocytes. Cells were washed then re-suspended in complete culture medium (RPMI 1640 media; Life Technologies, Burlington, ON, Canada), supplemented with 5% (v/v) heat-inactivated fetal calf serum, 25 mM HEPES, 2.5 mM 2-mercaptoethanol and 1% antibiotic/antimycotic (pH 7.4; Fisher Scientific, see above). Prior to *ex vivo* analyses, a haemocytometer was used to count live cells using trypan blue dye exclusion (Sigma-Aldrich, as above) to assess cell viability and was >90% for all treatment groups. All cell suspensions were then diluted to 1.25 × 10^6^ cells/ml.

### Plasma Metabolites Measurement

Blood glucose was measured in the non-fasting state from the saphenous vein of 12-weeks-old rats with a glucometer (Accu-chek, Roche, Switzerland). At the end of the study, blood was collected by cardiac puncture in tubes containing EDTA, dipeptidyl peptidase 4 inhibitor (EMD Millipore, MA), and Complete^®^ general protease inhibitor (Sigma) before being centrifuged at 3,000 g for 10 min to obtain plasma. Plasma adiponectin and leptin were measured by ELISA according to the manufacturer's instructions. Plasma cytokines and chemokines concentration were measured using a Proinflammatory Panel 1 V-PLEX electrochemiluminescent multiplex cytokine kit (Meso Scale Discovery, USA) to determine circulating concentrations of IFN- γ, IL-10, IL-13, IL-1β, IL-4, IL-5, IL-6, and TNF-α and chemokine KC/GRO (keratinocyte chemoattractant/human growth-regulated oncogene) following the manufacturer's instructions.

### Immune Cell Phenotype Analysis

Four multicolor flow cytometry panels were design and splenocytes were stained with different antibodies as follow: T-cell panel: CD4 (FITC), CD3(APC-Vio770), CD8 (BUV395), CD28 (PE-Vio770), CD25 (PercP-eFluor710), CD127 (AlexaFluor594), CD27 (BUV737); B cell panel: CD45RA (AlexaFluor40), CD80 (PE), CD27 (BUV737), CD74 (OX6, PerCP); Natural killer cells panel: CD161 (Alexa647), OX62 (PE), CD74 (OX6, PerCP), CD3 (APC-Vio770); Monocytes and macrophages panel: CD68 (APC-Vio770), CD11bc (PE-Vio770). The proportion of CD3+CD8- cells were considered as CD3+CD4+ cells because the CD4-fluorescein isothiocyanate did not stain properly. All panels contained viability dye (Zombie Yellow-V525). After incubation, cells were washed with PBS and fixed in paraformaldehyde (10 g/l; Thermo Fisher Scientific) in phosphate-buffered saline. Cell events were collected within 72 h of preparation on a BD LSRFortessa X20 SORP flow cytometer and data was analyzed using FlowJo v10 (USA). Regarding the gating strategy, lymphocyte populations were first gated based on forward scatter (FSC) (28) area vs. side scatter (SSC) area. Debris and doublets were then excluded, and viable cells were identified in all stained panels based on FSC area vs. FSC height. For live lymphocytes, total T-cells were defined as CD3+ and within CD3+ cells, helper and cytotoxic T-cells were then identified by gating on CD8– and CD8+, respectively. Helper and cytotoxic T-cells expressing IL-2 α chain receptor were identified as CD3+CD8–CD25+ and CD3+CD8+CD25+. An example of a gating strategy is presented in Supplementary File 1. B cells were identified as CD45RA+. Activated B cells were identified from CD45RA+ as CD45RA+CD80+. Macrophages were gated as CD68+. Within OX62+, dendritic cells were defined as OX62+OX6. Natural killer cells were identified as CD161+CD3-. The median fluorescence intensity (MFI) of CD25+ in CD3+, CD4+ and CD8+ was determined. Since there was no difference in the number of splenocytes between groups, only the proportion of immune cells are presented.

### *Ex vivo* Cytokine Production by Mitogen-Stimulated Cells

The measurement of cytokines production by mitogen-stimulated cells in spleen has been previously described (29). Briefly, cells (1.25 × 10^6^ cells/ml) were cultured in 3 ml RMPI-1640 medium (as above) for 48 h at 37°C and 5% CO_2_ without mitogen (unstimulated) or with mitogens including phorbol 12-myristate 13-acetate plus ionomycin (PMA+I; 2 μg/ml); lipopolysaccharide (LPS; 5 μg/ml); pokeweed (PWM; 55 μg/ml). PMA+I activates T-cells, LPS activates primarily antigen presenting cells (APC), and PWM activates T-cells and APC. After incubation, cells were centrifuged for 10 min at 1,000 rpm and supernatants collected and stored at −80°C until analyses. Concentrations of interleukin (IL)-1β, IL-2, IL-6, IL-10, tumor-necrosis factor-α (TNF-α), and interferon-γ (IFN-γ) were measured by commercial ELISA kits according to manufacturer's instructions (R&D Systems, Minneapolis, MN, USA) and as previously described (29). The detection limits for all cytokines were 15.6–4,000 pg/ml (R&D Systems, Minneapolis, MN, USA). Cytokine concentrations were quantified using a microplate reader (SpectraMax 190; Molecular Devices, Sunnyvale, CA, USA) and all measurements were conducted in duplicates, with CV < 10%. IL-1β was only measured in the supernatant of LPS and PWM stimulated cells and IL-2 was only measured in the supernatant of PMA+I and PWM stimulated cells.

### *Ex vivo* T-Cell Proliferation Assay

96-well plates were coated with 1 μg /mL of anti-CD3 and were incubated overnight at 4°C. Frozen splenocytes were warm thawed for 10 min at 37°C before being washed with DMSO in 10% RPMI 1640 medium (Life Technologies, Burlington, ON, Canada). Afterwards, cells were transferred in 5 mL 10% RPMI to 10 mL culture tubes and incubated for 4 h at 37°C. After incubating, cells were washed and resuspended in 10% RPMI. A haemocytometer was used to count live cells using trypan blue dye exclusion (Sigma-Aldrich) to assess cell viability. The 96-well plates were set up to get 1.25 × 10^6^ cells/mL in 200 uL. Samples were added in triplicate, with anti-CD28 stimulation (0.3 ug/mL) of previously coated anti-CD3 wells along with unstimulated cells (30). Plates were then incubated at 37°C for 72 h. After incubation, cell numbers were fixed with 100 uL 4% PFA for 20 min, followed by a 20 min incubation with crystal violet (Acros Organic) solution (0.1%, w/v, with ethanol 2%, v/v in 0.5 M Tris-C1, pH 7.8; 100 ul per well) at room temperature. The stained cell layer was rinsed thoroughly with deionized water, vacuum aspirated and incubated with sodium dodecyl sulfate (SDS) solution (0.1%, w/v, with ethanol 50%, v/v, in 0.5 M Tris-C1, pH 7.8; 100 uL per well) for 30 min at room temperature. Meanwhile, crystal violet was completely released from the cells into the supernatant. The supernatant was scanned in a microplate reader (SpectraMax 190; Molecular Devices, Sunnyvale, CA, USA) and read at a fixed wavelength of 590 nm. All samples were measured in triplicate. One male rat fed the CLF did not have enough viable cells to perform the assay and its data was excluded from the analysis.

### Statistical Analysis

Data are reported as mean ± standard error of the mean (SEM) unless indicated otherwise. Data was analyzed using one-way ANOVA in SAS (v9.4, Cary, NC) with diet as the main effect. The study was powered to assess significant differences in immune function (i.e., *ex vivo* cytokine production as the primary outcome) as main effect. In cases where a significant main effect was found, *post-hoc* analysis was performed using the DUNCAN adjustment to determine differences between diet groups. Variables that were not normally distributed were log-10 transformed prior to statistical analysis. Differences at *p* ≤ 0.05 (two-sided) were considered significant.

## Results

### Anthropometric Characteristics and Daily Food Intake

**Figure 1:**
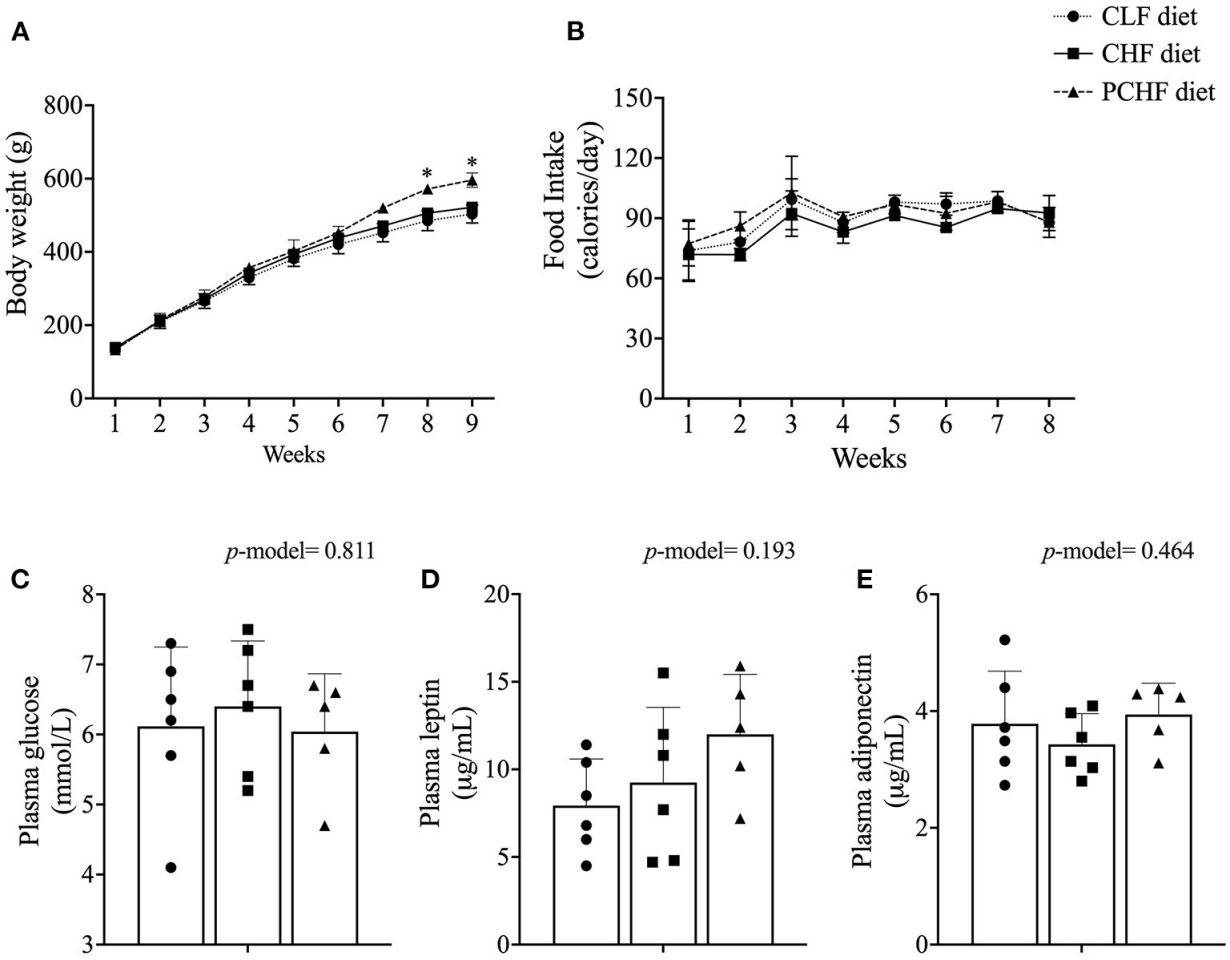
**(A)** Body weight; **(B)** caloric intake; **(C)** non-fasting plasma levels of glucose; **(D)** leptin and **(E)** adiponectin of male Wistar rats fed the three experimental diets. Values are presented as mean ± SEM. *Means significantly different (*p* < 0.05) from the CLF diet based on the one-way ANOVA test with the Duncan adjustment for multiple comparisons. CLF, control low-fat; CHF, control high-fat; PC, phosphatidylcholine; PCHF, PC high-fat.

Male Wistar rats fed the PCHF diet had a significantly higher body weight at the end of the feeding experiment when compared to the CLF diet (all *p* < 0.05, Figure 1A). No changes in body weight were found between the CLF and the CHF diets (all *p* > 0.05). Rats from the CLF group had a significant higher food intake (g/day) when compared to the CHF and PCHF groups (*p* < 0.05, Supplementary File 2). Consequently, the calorie intake per day was similar between the groups (Figure 1B). Animal and intestinal length, organ's weight and splenocytes count (all *p* > 0.05, Table 3) were all unaffected by the dietary treatments.

**Table 3:** Anthropometric and plasma cytokine data of male Wistar rats fed the three experimental diets^a^.

**Variables**	**CLF diet** **(***n*** = 6)**	**CHF diet** **(***n*** = 6)**	**PCHF diet** **(***n*** = 5)**	***p-*** **model**
Animal length (cm)	25.2 ± 0.31	25.0 ± 0.36	25.4 ± 0.51	0.779
Spleen weight (g)^b^	1.36 ± 0.12	1.49 ± 0.08	1.65 ± 0.04	0.166
Splenocytes, *n* × 10^6^/100 g tissue^b^	12.8 ± 3.22	7.62 ± 0.84	9.08 ± 1.33	0.230
Liver weight (g)	26.1 ± 1.33	25.7 ± 1.13	28.3 ± 1.30	0.325
Intestine length (cm)	123.2 ± 4.14	121.3 ± 3.14	125.6 ± 0.6	0.662
**Plasma cytokines**				
IL-13 (pg/mL)	1.81 ± 0.31	2.78 ± 0.27	1.83 ± 1.16	0.381
IL-1β (pg/mL)	32.4 ± 4.28	38.5 ± 8.88	18.3 ± 13.0	0.360
IL-4 (pg/mL)	1.67 ± 0.48	1.19 ± 0.24	0.98 ± 0.50	0.512
IL-5 (pg/mL)	110.9 ± 7.62	116.9 ± 13.9	83.4 ± 22.2	0.298
IL-6 (pg/mL)	58.3 ± 9.38	60.1 ± 11.2	37.0 ± 11.1	0.328
TNF-α (pg/mL)	10.6 ± 1.09	10.8 ± 1.24	9.69 ± 0.83	0.765
IL-10 (pg/mL)	16.1 ± 0.60	12.7 ± 2.53	12.9 ± 4.52	0.614
IFN-γ (pg/mL)	8.98 ± 2.13	8.09 ± 1.72	8.36 ± 4.64	0.965
KC/GRO (pg/mL)^b^	65.4 ± 19.8	54.6 ± 16.8	29.7 ± 5.03	0.345

a *Values are means ± SEM, n = 6. Groups that do not share the same letter are significantly different based on the one-way ANOVA test with the Duncan adjustment for multiple comparisons test (p < 0.05). CLF, control low-fat; CHF, control high-fat; IFN-γ, interferon gamma; IL, interleukin; KC/GRO, keratinocyte chemoattractant/human growth-regulated oncogene; PCHF, PC high-fat; PC, phosphatidylcholine; TNF-α, tumor necrosis factor alpha*.

b *Analysis performed on log-transformed values*.

### Plasma Metabolites

There was no significant difference in plasma glucose levels amongst dietary groups (all *p* > 0.05, Figure 1C). Plasma level of adiponectin and leptin were similar between groups (all *p* > 0.05, Figures 1D,E). In addition, no significant differences were found in the concentration of plasma cytokines and chemokines between groups (Table 3).

### Liver Total Fatty Acid Composition

Despite the fact that all three diets were closely matched in term of fatty acids composition, several changes in liver total fatty acid composition were noted (Supplementary File 3). The PCHF diet led to an increased proportion of docosapentaenoic acid (DPA) and total PUFA when compared to the CLF diet. Both the PCHF and CHF diets had a higher concentration of α-linolenic acid (ALA) and total n-6 PUFA and a lower proportion of total SFAs when compared to the CLF. The PUFA/SFA ratio was higher in PCHF when compared to both the CHF and CLF diets. The CHF had a higher PUFA/SFA ratio compared to the CLF. No changes were found in the proportion of arachidonic acid (AA) or docosahexaenoic acid (DHA) among groups.

### T-Cell Proliferation Assay and *ex-vivo* Cytokine Production by Splenocytes After Mitogen Stimulation

**Figure 2:**
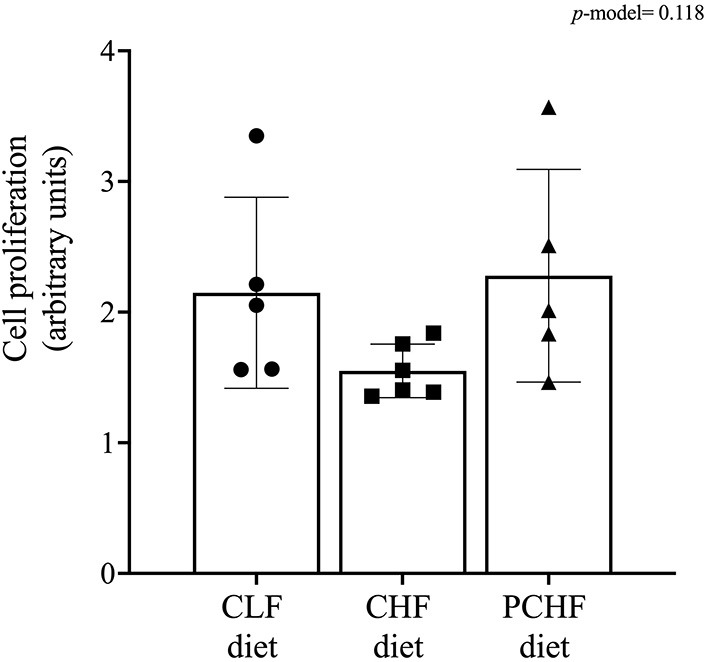
Effect of diet on T-cell proliferation following stimulation with anti-CD3/anti-CD28 in male Wistar rats. Values are means ± SEM. *p*-model = 0.118. CLF, control low-fat; CHF, control high-fat; PC, phosphatidylcholine; PCHF, PC high-fat.

Diet had little impact on T-cell proliferation by splenocytes after stimulation with anti-CD3/anti-CD28 (*p* = 0.118) as shown in Figure 2. *Ex-vivo* cytokine production by splenocytes isolated from male Wistar rats is presented in Table 4. After PMA+I stimulation, rats fed with the CHF diet had a significantly lower production of IL-2 and TNF-α when compared to the CLF and PCHF diets (both *p* < 0.05) and a trend toward a lower IL-6 production when compared to the CLF diet only (*p* = 0.055). No changes were observed in IFN-γ and IL-10 production between groups (both *p* > 0.05). Following LPS stimulation, there was no significant change in cytokine production among diet groups (all *p* > 0.05). After PWM stimulation, there was an increased production of IL-6 in the CHF group when compared to the CLF group, although it did not reach significance (*p* > 0.05). There were no significant differences between the PCHF and the CLF groups after PWM stimulation (all *p* > 0.05).

**Table 4:** *Ex vivo* cytokine production by mitogen-stimulated splenocytes of male Wistar rats fed the three experimental diets.

**Variable^1^**	**CLF diet** **(***n*** = 6)**	**CHF diet** **(***n*** = 6)**	**PCHF diet** **(***n*** = 5)**	***p-*** **model**
**PMA+I (T-cell mitogen)**				
IL-2 (pg/ml)	2,146.4 ± 173.2^a^	883.3 ± 223.4^b^	2,246.8 ± 385.0^a^	0.005
IL-10 (pg/ml)	166.8 ± 37.9	207.6 ± 24.6	225.6 ± 13.9	0.407
TNF-α (pg/ml)	102.1 ± 13.8^a^	61.2 ± 8.5^b^	131.5 ± 12.8^a^	0.004
IL-6 (pg/ml)	220.1 ± 52.8^a^	54.1 ± 18.7^b^	137.7 ± 46.8^ab^	0.055
**LPS (bacterial challenge)**				
IL-1β (pg/ml)	28.0 ± 4.68	33.5 ± 5.27	34.4 ± 3.93	0.595
IL-10 (pg/ml)	487.8 ± 110.3	569.9 ± 75.6	663.0 ± 42.9	0.445
TNF-α (pg/ml)^2^	189.0 ± 18.9	172.7 ± 21.7	226.0 ± 28.7	0.288
IL-6 (pg/ml)	518.9 ± 57.1	565.7 ± 82.6	592.6 ± 90.5	0.814
**PWM (T and APC cells mitogen)**				
IL-1β (pg/ml)	110.6 ± 13.24	152.9 ± 34.3	123.8 ± 25.6	0.537
IL-2 (pg/ml)	432.8 ± 71.0	357.6 ± 74.6	425.9 ± 26.7	0.673
IL-10 (pg/ml)	385.2 ± 52.8	527.2 ± 61.6	514.0 ± 66.6	0.199
TNF-α (pg/ml)^2^	311.8 ± 45.8	268.0 ± 31.9	398.8 ± 68.1	0.536
IFN-γ (pg/ml)^2^	119.0 ± 49.3	315.5 ± 172.6	311.9 ± 199.2	0.200
IL-6 (pg/ml)	1,258.6 ± 215.6^b^	2,043.7 ± 323.0^a^	1,612.7 ± 149.6^ab^	0.109

1 *Values are means ± SEM. Groups that do not share the same letter are significantly different based on the one-way ANOVA test with the Duncan adjustment for multiple comparisons test (p < 0.05). CLF, control low-fat; CHF, control high-fat, PCHF, PC high-fat; PC, phosphatidylcholine; IFN-γ, interferon gamma; IL, interleukin; TNF-α, tumor necrosis factor alpha*.

2 *Analysis performed on log-transformed values*.

### Splenocyte Phenotypes

Proportions of immune cell phenotypes of male Wistar rats fed the three experimental diets are presented in Table 5. Rats fed with the PCHF diet had a lower proportion of CD3+ cells when compared to both the CLF and CHF diets (*p* < 0.05). There were no significant differences in the proportion of helper T-cells (CD3+CD4+) and cytotoxic T-cells (CD3+CD8+) as well as activated T-cells (CD3+CD25+) among groups (both *p* > 0.05). The MFI of CD25+ in CD4+ was higher in the CHF when compared to the CLF group only. No changes were found in the MFI of CD25+ in CD3+ and CD8+. The proportions of total CD127+ cells were lower in the PCHF diet when compared to the CHF diet. No significant changes were found in the proportion of B cells between groups nor in the proportion of activated B cells (CD45RA+CD80+). In addition, no significant differences in the proportion of CD28+ (co-stimulatory molecule), CD27+ (memory marker), natural killer cells (NK, CD3-CD161+) and macrophages (CD11bc+), OX6+ (MHC II cells) were noted.

**Table 5:** Splenocyte phenotype of male Wistar rats fed the three experimental diets.

**Phenotype^10^**	**CLF diet** **(***n*** = 6)**	**CHF diet** **(***n*** = 6)**	**PCHF diet** **(***n*** = 5)**	***p-*** **model**
**% of total gated cells**				
Total CD3+ (T-cells)	38.9 ± 1.94^a^	36.9 ± 1.54^a^	31.7 ± 1.19^b^	0.024
**% of CD3+** **gated cells**				
CD4+ (helper T-cells)	80.9 ± 0.91	78.7 ± 1.68	79.1 ± 1.96	0.556
CD8+ (cytotoxic T-cells)	20.1 ± 1.66	23.1 ± 2.79	20.2 ± 2.24	0.575
CD25+ (activated T-cells)^2^	14.0 ± 1.39	15.9 ± 1.10	16.7 ± 0.56	0.280
CD4+CD25+	23.2 ± 2.37	22.7 ± 3.01	20.8 ± 2.30	0.814
CD8+CD25+	16.6 ± 1.42	18.4 ± 1.33	20.5 ± 0.67	0.137
**% of total gated cells**				
Total CD28+ (Co-stimulatory molecule)	19.9 ± 4.90	31.5 ± 4.22	23.8 ± 4.30	0.235
Total CD127+	4.03 ± 0.31^ab^	4.36 ± 0.41^a^	3.05 ± 0.42^b^	0.079
Total CD80+	11.3 ± 0.71	10.4 ± 0.70	11.1 ± 1.30	0.758
Total CD45RA+ (B cells)	35.5 ± 2.05	38.9 ± 1.81	31.5 ± 5.40	0.411
CD45RA+CD80+	10.6 ± 0.91	10.2 ± 1.15	11.1 ± 1.26	0.839
Total CD68+ (macrophages)^2^	9.55 ± 0.90	9.48 ± 0.42	7.90 ± 1.12	0.319
Total OX6+ (MHC class II+)	40.8 ± 1.16	42.2 ± 3.30	39.5 ± 4.98	0.851
Total OX62+	10.7 ± 1.63	11.6 ± 2.00	9.92 ± 2.00	0.818
OX62+OX6+ (dendritic cells)	1.82 ± 0.31	2.78 ± 0.27	1.83 ± 1.16	0.381
CD161+CD3- (natural killer cells)	9.36 ± 0.64	11.2 ± 0.89	10.8 ± 1.16	0.317
**CD25+** **MFI**				
CD3+	2,829.5 ± 296.9	3,364.3 ± 300.7	2,595.6 ± 270.3	0.202
CD4+	2,280.2 ± 238.9^b^	3,099.5 ± 117.4^a^	2,573.2 ± 280.6^ab^	0.045
CD8+^2^	1,105.7 ± 242.0	1,147.8 ± 121.1	1,060.8 ± 172.5	0.845

1 *Values are presented as mean ± SEM. Groups that do not share the same letter are significantly different based on the one-way ANOVA test with the Duncan adjustment for multiple comparisons test (p < 0.05). CD3+CD8-CD28+ (average 58.5 ± 13.6); CD3+CD8+CD28+ (average 71.9 ± 3.80); CD3+CD8-CD127+ (4.60 ± 0.77); CD3+CD8+CD127+ (6.17 ± 0.82); Total CD11bc+ (average 16.0 ± 1.43). CLF, control low-fat; CHF, control high-fat, MFI, median fluorescence intensity; PCHF, PC high-fat; PC, phosphatidylcholine*.

2 *Analysis performed on log-transformed values*.

## Discussion

In this study, we aim to determine the effect of egg-PC supplementation on immune function in male Wistar rats fed a HFD. Our data support our hypothesis that feeding a HFD impairs immune responses and that adding PC as part of a HFD attenuates the obesity-related T-cell dysfunction. Of note, this is the first study where the proportion of different fatty acid in the diets were matched between the low-fat and high-fat diets to assess immune responses in this model. This is crucial since fatty acids are known to influence immune function in that saturated and omega-3 polyunsaturated fatty acids exert pro- and anti-inflammatory properties, respectively (31, 32). Therefore, matching the proportion of fatty acids when assessing the effect of choline forms (water vs. lipid-soluble) on the immune system is important. Despite diets being matched for the fatty acid composition, in liver the PCHF diet led to a lower proportion of SFAs and a higher proportion of DPA, PUFAs and an overall higher PUFA/SFA ratio. A higher PUFA/SFA ratio has been associated with a lower risk of cardiovascular disease (33).

We first demonstrated that providing a HFD with PC increased rats' body weight compared to the CLF at the end of the feeding experiment (weeks 8 and 9). We previously showed that feeding PC and other lipid soluble form of choline early in life improve pups' growth (19). In order to be absorbed by the enterocyte, PC is first broken down to Lyso-PC by Phospholipase A2 and once in the enterocyte can be reacetylated to PC or further be broken down into GPC (34). GPC has been shown to increased plasma levels of growth hormone, which can increases growth, tissue regeneration, and cell production (35). The CHF diet had no impact on body weight compared to the CLF diet through the duration of the experiment. This is mainly attributable to the same caloric intake by the CLF group when compared to the HFD diets.

We demonstrated that feeding a HFD containing only FC impairs T-cell responses in male Wistar rats after PMA+I stimulation by decreasing the production of IL-2 and TNF-α, a Th1 pro-inflammatory cytokine. In addition, rats fed the HFD had a lower T-cell proliferation rate after stimulation with anti-CD3/anti-CD28, although it did not reach significance (*p* = 0.118). These results are consistent with previous studies where feeding Wistar rats with a cafeteria diet also impaired IL-2 production after ConA stimulation (a T-cell mitogen) (10). We also showed that the CHF group produced less IL-2 despite having an increase expression of CD25 in helper T-cells as measured by MFI. This is consistent to some extent with a previous study from our group where we demonstrated that PBMCs isolated from individuals with obesity and T2D produced less IL-2, TNF-α, and IL-6 when stimulated with phytohemagglutinin (another T-cell mitogen) than individuals with obesity who were metabolically healthy and this, despite having a higher proportion of helper T-cell expressing CD278 which plays a role in IL-2 synthesis and T-cell proliferation (3, 36). The underlying mechanism as to how PC modulates T-cell function is still unclear. PC, as a lipid-soluble form of choline, is mostly absorbed and secreted through chylomicrons and enter the bloodstream first to be potentially delivered to peripheral organs (i.e., including the spleen) instead of reaching the portal vein first and going to the liver (34). Feeding a diet containing primarily PC could potentially affect the incorporation of PC into cell membranes (i.e., splenocytes) and therefore modulate its composition. In that regard, we have demonstrated that a diet containing a 100% PC during the suckling period increased the proportion of PC in splenocyte cell membranes of suckled pups (18). How the incorporation of PC into the lipid rafts occurs which might affect immune responses remains to be investigated. In addition, *in vitro* studies by our group have shown that incubating splenocytes with Lyso-PC (the form of PC readily available to cell) increased the proliferation rate and IL-2 production as measured by water-soluble tetrazolium salt assay, suggesting that PC can indeed modulate T-cell function (18).

In our previous studies we have noted that PC supplementation early in life primarily affects T-cell function and to a lesser extent B cell function. Moreover, in humans, we have also reported that obesity and T2D affect particularly T-cells and neutrophil function, while no effect on B cells function was observed as measured by *ex vivo* cytokine production after LPS stimulation. Consistent with previous study, we showed that a HFD primarily affects T-cells function whereas no changes were seen in APC (B cells, monocytes, and dendritic cells) function. Although obesity is associated with a higher proportion of pro-inflammatory M1-like macrophages in adipose tissue (37), the proportion of circulating B cell, monocyte and NK cells appears to be unaltered as reported in some studies (3, 38). In the current study, we did not find significant differences in the frequency of monocytes/macrophages (CD68+), B cells (CD45RA+), activated B cells (CD45RA+CD80+), OX6+ cells (MHC II), dendritic cells (OX6+OX62+) or NK cells (CD3-CD161+) in spleen among groups.

Chronic systemic low-grade inflammation is characterized by elevated circulating levels of CRP, TNF-α and IL-6 (39). Adipose tissue appears to be an important contributor to systemic inflammation and its related metabolic complications such as insulin resistance. Indeed, M1-like macrophages are known to promote the secretion of cytokines such as TNF-α and IL-6 (37) and both IL-6 and TNF-α have been linked to insulin resistance by impairing insulin receptor signaling (40, 41). Insulin resistance is thought to play a key role in modulating immune cell responses since immune cells that lack the insulin receptor have been shown to have an impaired cytokine secretion and lower antigen presentation activity upon influenza infection in a rodent model (42). High-fat feeding has also been reported to promote intestinal inflammation and gut permeability, which can contribute to systemic inflammation (43). In addition, high-fat feeding has also been associated with higher leptin and lower adiponectin concentrations in rodents (44, 45). Yet in our study, plasma cytokine levels were unaffected by dietary treatments along with non-fasting glucose and leptin and adiponectin concentrations, although we observed trends toward an “obesity phenotype”. This could be explained at least in part by the fact that rats fed the CLF diet were consuming slightly more g/day of diet and that no differences in body weight were observed between the CLF and the CHF group. Pair-feeding the animal on the CLF to the ones on the HF diets would have most likely led to differences in those parameters. Another explanation could be that all our diets were matched closely for the proportion of fatty acids whereas most diets used to induce obesity are higher in SFAs compared to typical low-fat diets. Previous studies have shown that SFAs, such as palmitic acid and lauric acid, are capable of initiating an inflammatory response by binding to Toll-like receptor 4 (TLR-4) (46). Finally, glucose measurements were done in the non-fasting state and since the CLF diet was much higher in carbohydrate, this could explain the similar plasma glucose values between groups. Overall, our results suggest that the changes observed in T-cell function in the CHF group are likely attributable to other mechanisms which could include increased gut permeability and increased bacterial translocation.

Although our study has strengths which include the design of our diets being closely matched for choline content and fatty acid composition and the assessment of several factors that have been proposed to be involved in modulating immune function, some limitations should also be stressed. Although our sample size was enough to see differences in our primary outcome (i.e., *ex vivo* cytokine production), we might have been underpowered for some of our secondary analysis with higher inter-individual variations. Other potential mechanism, including gut permeability, were not assessed which could have provided a better understanding of how PC modulates immune function in the context of a HFD. Moreover, body composition was not measured in this study to determine if there were differences in body fat mass regardless of the similarities in body weight between our diet groups. In addition, a diet containing 100% PC is not physiologically relevant to human consumption, therefore, a dose-response study is needed in order to establish the optimal proportion of PC to modulate T-cell responses while being of relevance for human consumption. Finally, although *ex vivo* cytokine production of different mitogens provides an overall understanding of how different immune cells populations respond to a challenge, other methods such as intracellular analysis of cytokines by flow cytometry could provide a better understanding of specific immune cells population response.

## Conclusion

In summary, our results suggest that feeding a HFD impairs T-cells function while having little effect on antigen-presenting cells function in male Wistar rats. We showed that providing egg-PC as part of a HFD can normalize T-cell responses, as measured by *ex vivo* cytokine production, to some extent in Wistar rats by increasing the production of IL-2 and Th1 cytokines, known to promote T-cell proliferation.

## Data Availability Statement

The original contributions presented in the study are included in the article/Supplementary Material, further inquiries can be directed to the corresponding author/s.

## Ethics Statement

The animal study was reviewed and approved by the University of Alberta Animal Ethics Committee.

## Author Contributions

CR and RJ designed and obtained funding for this study. SG, AM, JA-B, BW, and HV-S conducted research and analyzed data. JA-B performed the statistical analysis and wrote the manuscript under the supervision of CR, RJ, and CF. CR has primary responsibility for final content. All authors have read and approved the final manuscript.

## Funding

This study was supported by grants from the Natural Sciences and Engineering Research Council of Canada (RES0038933) and Egg Farmers of Canada (RES0046255). JA-B was a recipient of a PhD CONACYT (Consejo Nacional de Ciencia y Tecnología) scholarship from Mexico.

## Conflict of Interest

The authors declare that the research was conducted in the absence of any commercial or financial relationships that could be construed as a potential conflict of interest.

## Publisher's Note

All claims expressed in this article are solely those of the authors and do not necessarily represent those of their affiliated organizations, or those of the publisher, the editors and the reviewers. Any product that may be evaluated in this article, or claim that may be made by its manufacturer, is not guaranteed or endorsed by the publisher.

## Abbreviations

ALA: α-linolenic acid
APCs: antigen presenting cells
CLF: control low-fat
CHF: control high-fat
CRP: C-reactive protein
DHA: docosahexaenoic acid
FSC: forward scatter
GC: gas chromatography
HFD: high-fat diet
IFN-γ: interferon-gamma
IL: interleukin
KC/GRO: keratinocyte chemoattractant/human growth-regulated oncogene
LA: linoleic acid
LPS: lipopolysaccharide
MFI: median fluorescence intensity
MRI: magnetic resonance imaging
MUFA: monounsaturated fatty acid
n: omega
NK: natural killer
PC: phosphatidylcholine
PCHF: PC high-fat
PerCP: peridinin chlorophyll protein
PBMCs: peripheral blood mononuclear cells
PE: phycoerythrin
PMA+I: phorbol 12-myristate 13-acetate plus ionomycin
PWM: pokeweed mitogen
PUFA: polyunsaturated fatty acid
RBC: red blood cells
RDW: red cell distribution
SFA: saturated fatty acid
SEM: standard error of the mean
SSC: side scatter
T2D: type 2 diabetes
Th1: T helper 1
Th2: T helper 2
TNF-α: tumor necrosis factor-alpha.
