# Corrigendum: Egg-phosphatidylcholine attenuates T-cell dysfunction in high-fat diet fed male Wistar rats

**DOI:** 10.3389/fnut.2023.1304098

**Published:** 2023-10-11

**Authors:** Jessy Azarcoya-Barrera, Bethany Wollin, Hellen Veida-Silva, Alexander Makarowski, Susan Goruk, Catherine J. Field, René L. Jacobs, Caroline Richard

**Affiliations:** Department of Agricultural, Food and Nutritional Science, University of Alberta, Edmonton, AB, Canada

**Keywords:** high-fat diet, phosphatidylcholine, immunology, obesity, egg

In the published article, there was an error in the **Acknowledgements** statement. Support received by CR from the Canada Research Chair program was omitted from the statement.

The corrected Acknowledgements statement appears below.

## Acknowledgments

The authors would like to acknowledge the technical assistance of Dr. Aja Rieger, Mariana Juarez-Platas, Dhruvesh Patel, Nicole Coursen, Marnie Newell, and Audric Moses. We express our gratitude to the undergraduate students that worked on the project. CR was supported by the Canada Research Chair program (CRC-2018-00081).

The authors apologize for this error and state that this does not change the scientific conclusions of the article in any way. The original article has been updated.

