# Supplementary material for: Egg-Phosphatidylcholine Attenuates T-Cell Dysfunction in High-Fat Diet Fed Male Wistar Rats
Source: Front Nutr. 2022 Feb 2;9:811469. doi: 10.3389/fnut.2022.811469 (PMC8847771; doi:10.3389/fnut.2022.811469)

Supplementary File 1. Gating strategy. (**A**) Lymphocyte population based on forward scatter area versus side scatter area; (**B**) Single cells selected, doublets excluded; (**C**) cells alive were identified using viability dye (Zombie Yellow-V525); (**D**) total T cells population identified as CD3+ within the live lymphocytes population; (**E**) cytotoxic T cells were gated within the CD3+ population and identified as CD8+ cells. SSC-A, side scatter area; FSC-A, forward scatter area; FSC-H, forward scatter height.

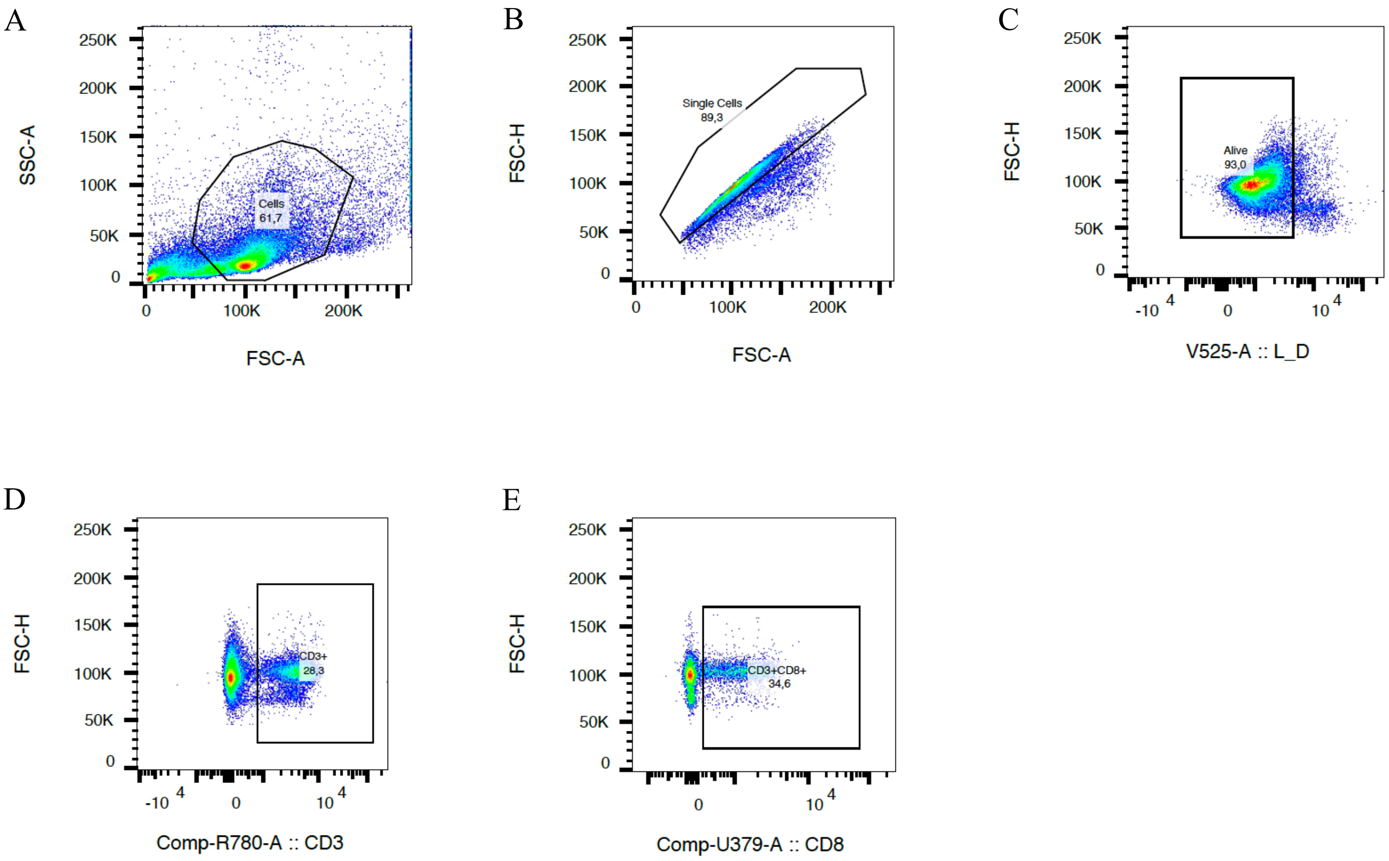

Supplement: Supplementary file 1 [file Data_Sheet_1.PDF]
