# Supplementary material for: Egg-Phosphatidylcholine Attenuates T-Cell Dysfunction in High-Fat Diet Fed Male Wistar Rats
Source: Front Nutr. 2022 Feb 2;9:811469. doi: 10.3389/fnut.2022.811469 (PMC8847771; doi:10.3389/fnut.2022.811469)

Supplementary File 2. Food intake in g/day of male Wistar rats fed the three experimental diets. Values are presented as mean ± SEM. * CLF significantly different ($p < 0.05$) from both the CHF and the PCHF diet; † CLF significantly different from the CHF diet only based on the one-way ANOVA test with the Duncan adjustment for multiple comparisons performed at each time point. CLF, control low-fat; CHF, control high-fat; PCHF, PC high-fat; PC, phosphatidylcholine.

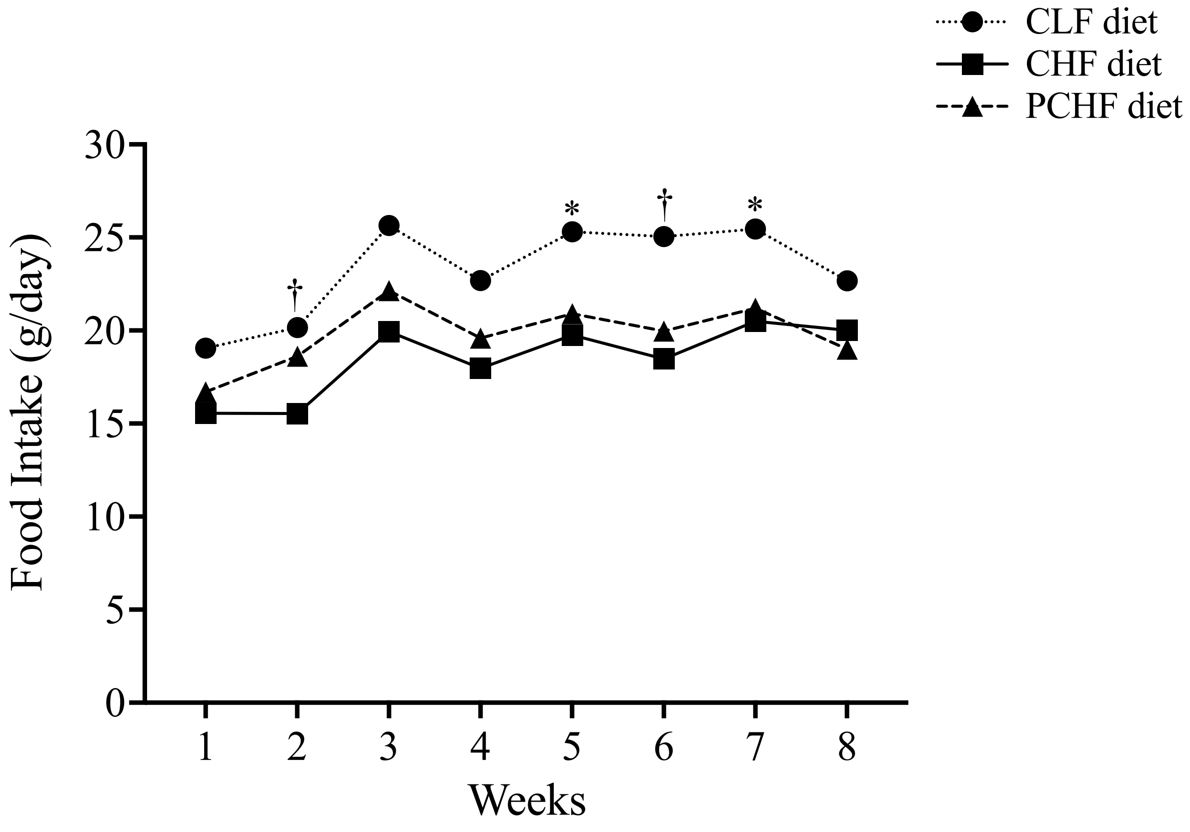

Supplement: Supplementary file 2 [file Data_Sheet_2.PDF]
