# Supplementary material for: Egg-Phosphatidylcholine Attenuates T-Cell Dysfunction in High-Fat Diet Fed Male Wistar Rats
Source: Front Nutr. 2022 Feb 2;9:811469. doi: 10.3389/fnut.2022.811469 (PMC8847771; doi:10.3389/fnut.2022.811469)
Supplement: Supplementary file 3 [file Data_Sheet_3.PDF]

Supplementary File 3. Liver total lipids fatty acid composition in male Wistar rats fed the three experimental diets.

Fatty Acid	CLF diet (n=6)	CHF diet (n=6)	PCHF diet (n=5)	*p* model
	(% of total fatty acids)			
14:0	0.51 ± 0.07[a]	0.39 ± 0.03[b]	0.33 ± 0.03[b]	0.081
14:1	0.19 ± 0.02[a]	0.15 ± 0.01[ab]	0.13 ± 0.01[b]	0.068
16:0	22.7 ± 0.64[a]	20.4 ± 0.33[b]	19.9 ± 0.69[b]	0.008
16:1n9	0.33 ± 0.02	0.41 ± 0.03	0.36 ± 0.03	0.173
16:1n7	1.59 ± 0.28[a]	0.75 ± 0.06[b]	0.69 ± 0.14[b]	0.005
17:0	0.30 ± 0.01[a]	0.26 ± 0.02[a]	0.20 ± 0.01[b]	0.003
18:0	19.1 ± 1.06	17.4 ± 0.61	16.3 ± 1.66	0.277
18:1n9	13.4 ± 1.28	17.0 ± 0.95	15.7 ± 2.35	0.297
18:1n7	2.50 ± 0.14[a]	1.37 ± 0.09[b]	1.20 ± 0.10[b]	<.001
18:2n6	13.9 ± 0.37[b]	18.5 ± 0.72[a]	20.1 ± 1.64[a]	0.003
20:0	0.15 ± 0.02	0.14 ± 0.03	0.13 ± 0.04	0.939
18:3n3 (ALA)	0.90 ± 0.07[b]	1.48 ± 0.13[a]	1.81± 0.26[a]	0.009
20:2n6	0.36 ± 0.02[b]	0.43 ± 0.02[ab]	0.44 ± 0.03[a]	0.060
20:3n6	0.66 ± 0.06	0.62 ± 0.04	0.76 ± 0.09	0.284
20:4n6 (AA)	16.8 ± 1.22	14.9 ± 0.73	15.2 ± 2.13	0.612
20:5n3 (EPA)	0.33 ± 0.04	0.40 ± 0.03	0.43 ± 0.01	0.155
24:0	0.01 ± 0.01	0.01 ± 0.0	0.01± 0.0	0.481
24:1n9	0.30 ± 0.02[b]	0.38 ± 0.02[ab]	0.49 ± 0.07[a]	0.017
22:4n6	0.11 ± 0.01[a]	0.07 ± 0.01[b]	0.12 ± 0.01[a]	0.002
22:5n6	0.02 ± 0.00[a]	0.01 ± 0.00[b]	0.01± 0.00[b]	0.003
22:5n3 (DPA)	0.63 ± 0.09[b]	0.81 ± 0.08[ab]	0.98 ± 0.10[a]	0.062
22:6n3 (DHA)	5.23 ± 0.52	4.17 ± 0.27	4.73 ± 0.64	0.320
Total SFA	42.8 ± 0.68[a]	38.6 ± 0.62[b]	36.9 ± 1.14[b]	0.001
Total MUFA	18.3 ± 1.50	20.1 ± 0.94	18.6 ± 2.49	0.728
Total PUFA	38.9 ± 1.46[b]	41.3 ± 0.85[ab]	44.6 ± 1.52[a]	0.029
Total n-6 PUFA	31.8 ± 0.90[b]	34.5 ± 0.59[a]	36.6 ± 1.05[a]	0.006
Total n-3 PUFA	7.10 ± 0.57	6.86 ± 0.29	7.95 ± 0.50	0.242
Ratio n-6/n-3	4.56 ± 0.24	5.06 ± 0.16	4.65 ± 0.17	0.162
Ratio PUFA/SFA	0.91 ± 0.04[c]	1.07 ± 0.03[b]	1.21 ± 0.03[a]	0.001

[1]Values are means ± SEM. Groups that do not share the same letter are significantly different based on the one-way ANOVA test with the Duncan adjustment for multiple comparisons test ($p < 0.05$). AA, arachidonic acid; ALA, α-linolenic acid; CLF, control low fat; CHF, control high-fat; DHA, docosahexaenoic acid; DPA, docosapentaenoic acid; EPA, eicosapentaenoic acid

MUFA, monounsaturated fatty acids; PCHF, PC high-fat; PC, phosphatidylcholine; PUFA, polyunsaturated fatty acids; SFA, saturated fatty acids.
